# Multi-Modal Mass Spectrometric Imaging of Uveal Melanoma

**DOI:** 10.3390/metabo11080560

**Published:** 2021-08-23

**Authors:** Laura M. Cole, Joshua Handley, Emmanuelle Claude, Catherine J. Duckett, Hardeep S. Mudhar, Karen Sisley, Malcolm R. Clench

**Affiliations:** 1 Centre for Mass Spectrometry Imaging, Biomolecular Sciences Research Centre, Sheffield Hallam University, Sheffield S1 1WB, UK; joshua.handley9@gmail.com (J.H.); c.duckett@shu.ac.uk (C.J.D.); m.r.clench@shu.ac.uk (M.R.C.); 2 Waters Corporation, Wilmslow SK9 4AX, UK; emmanuelle_claude@waters.com; 3 NSOPS, Department of Histopathology, E-Floor, Royal Hallamshire Hospital, Sheffield S10 2JF, UK; hardeep.mudhar@nhs.net; 4 Department of Oncology and Metabolism, The Medical School, University of Sheffield, Sheffield S10 2RX, UK; k.sisley@sheffield.ac.uk

**Keywords:** uveal melanoma mass spectrometry imaging, matrix assisted laser desorption ionisation, laser ablation inductively coupled plasma, cancer progression, genetic biomarkers, heterogeneity

## Abstract

Matrix assisted laser desorption ionisation mass spectrometry imaging (MALDI-MSI), was used to obtain images of lipids and metabolite distribution in formalin fixed and embedded in paraffin (FFPE) whole eye sections containing primary uveal melanomas (UM). Using this technique, it was possible to obtain images of lysophosphatidylcholine (LPC) type lipid distribution that highlighted the tumour regions. Laser ablation inductively coupled plasma mass spectrometry images (LA-ICP-MS) performed on UM sections showed increases in copper within the tumour periphery and intratumoural zinc in tissue from patients with poor prognosis. These preliminary data indicate that multi-modal MSI has the potential to provide insights into the role of trace metals and cancer metastasis.

## 1. Introduction

Uveal melanoma (UM) is the most common primary intraocular malignancy in adults, particularly amongst the Caucasian population [1]. The majority of patients develop UM within the choroid region, whilst others develop from the ciliary body or iris. UM metastases invade distant sites hematogenously, and metastatic spread usually targets the liver, but can also affect the lungs, skin and bones [2]. Approximately 50% of UM will metastasize and genetic biomarkers including monsomy 3 (M3) and gain of the long arm of chromosome 8 (+8q) are highly predictive of a poor outcome [3,4,5]. Less is however known about the relationship of genetic changes and the chemical biological information. Tumour biopsies or surgically removed tumours are routinely formalin fixed and embedded in paraffin (FFPE) for immediate histological analysis or retained archiving. The FFPE protocol has been extensively used for tissue preservation based on the methylene bridge cross linking properties [6,7]. Preparing FFPE tissues for mass spectrometry imaging presents many challenges, some of which include detachment of tissue from the microscope slide, interference from remaining paraffin wax, delocalisation and loss of target species of interest. Mass spectrometry imaging offers a powerful insight into the chemical biology of diseased tissue in the form of multiplexing experimentation where numerous species can be viewed within a single experiment in contrast to conventional histological staining. Observation of mass spectrometry profiles and images can provide invaluable links to and biological insights into whole disease pathways and networks. MALDI-MSI is a powerful molecular screening tool and can provide a distinctive molecular snapshot of diseased tissue. Additionally, these technologies allow conservation of the spatial distribution of a target species of interest. One key advantage of LA-ICP-MSI in contrast to MALDI-MSI is that there is no matrix requirement for sample ionisation which can help to minimise interference in the observation of images from small molecules. The following preliminary data reports the use of MALDI-MSI to investigate the retention of small biological molecules in FFPE whole eye sections containing primary UM. In addition to mapping the spatial distribution of molecular species that are of potential interest, trace metal analysis was also performed using LA-ICP-MSI to observe copper and zinc within enucleated eyes to provide information on the atomic pathology containing primary UM.

## 2. Results

**Figure 1:**
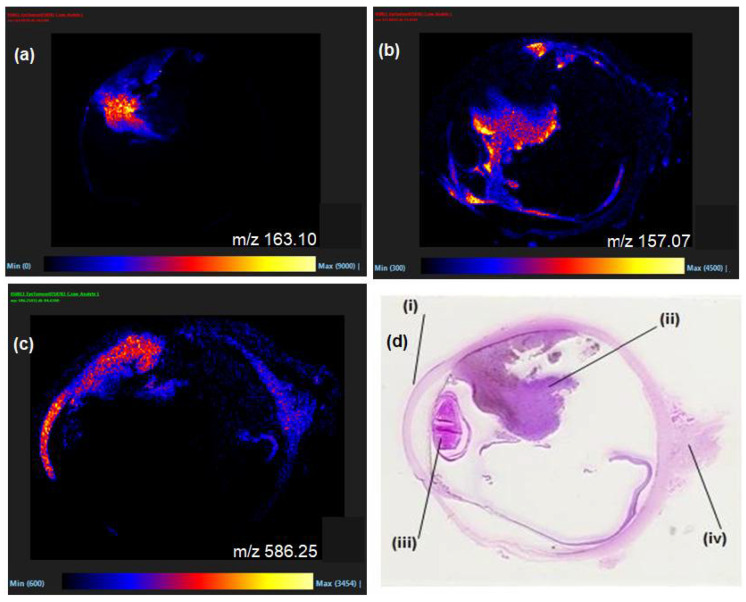
(**a**–**c**) Selected MALDI-MSI images of a FFPE section of human eye exhibiting UM with spatial distribution of *m/z* 586.2, [M + K]^+^, LPC 20:2 in (**c**), (**d**) H&E stained sectioned with (i) cornea, (ii) UM tumour regions, (iii) lens and (iv) optic nerve regions labelled.

Following dewaxing and antigen retrieval procedures, FFPE whole eye sections containing primary UM were examined by MALDI-MSI and provided an excellent opportunity to observe the distributions of species within the different tissues of an organ. Representative mass spectral images obtained by MALDI-MSI are displayed in Figure 1a–c along with the corresponding H&E-stained section (Figure 1d). These show the diverse spatial distribution of species throughout the various anatomical regions of the eye (i.e., the choroid, cornea, retina, lens and UM tumour regions). As can be seen, numerous species within the small molecule mass range were observable in the MALDI imaging experiments even after the many wash steps required for FFPE removal, demonstrating it to be a feasible method to analyse and process different tissues with the same protocol.

**Figure 2:**
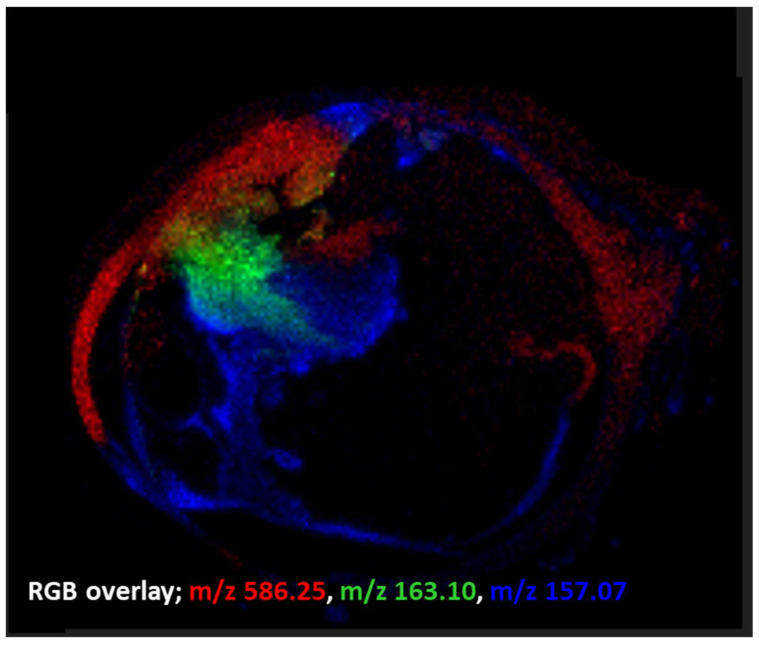
MALDI-MSI ion overlay data from an FFPE human eye section (merged from Figure 1 ion images). These data indicate that even after the many wash steps in FFPE removal and a standard antigen retrieval procedure, important low molecular weight species in tumour progression (e.g., *m/z* 586.2, [M + K]^+^, LPC 20:2) are still observable in MALDI-MSI. Images indicate distinct intratumoural heterogeneity.

Examples of ion image overlays employing the MALDI imaging technique used within this preliminary study can be seen in Figure 2.

**Figure 3:**
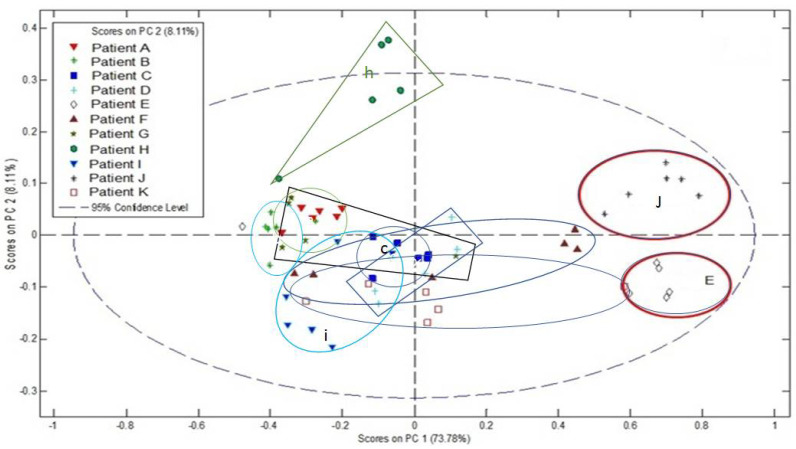
PCA analysis of tumour region MALDI-MSP data, from sections of FFPE human eye containing UM from 11 patients.

MALDI-MS profile data was acquired from the tumour regions of 11 separate sections of FFPE human eye tissue containing UM over the range *m/z* 100–1000. The results of PCA analysis of these data are shown in Figure 3. Sections were from excised eyes from 11 separate patients.

Clinical pathological details of the 11 patients are given in Table 1. The results of genetic biomarker analysis for each patient are also reported. Here, initial prognosis was determined by Fluorescence In Situ Hybridisation (FISH) analysis for copy number changes of chromosomes 3 and 8 and M3 and 8q+ was indicative of a very poor outcome as reported previously [8]. For some cases additional information was obtained by aCGH and further stratified the genetic biomarker classification [9]. There was no clear correlation with clinical or pathological markers, but patients who were determined as having a very poor outcome by genetic biomarkers tended to have more variance between readings and less clustering of data. 

**Figure 4:**
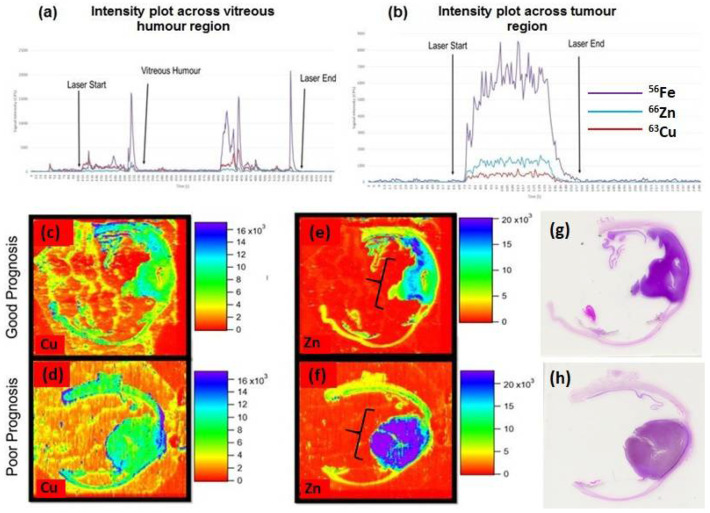
Intensity plots to show counts per second of ^63^Cu, ^66^Zn and ^56^Fe across one line of (**a**) whole eye section and (**b**) UM tumour region, acquired in KED mode. Spatial elemental distribution of elements in uveal melanoma sections, recorded as counts per second (CPS). (**c**,**d**) display copper (^63^Cu) distribution of good and poor prognosis, respectively (see patient E (good) and H (very poor) in Table 1), (**e**,**f**) show zinc (^66^Zn) distribution of good and poor prognosis, respectively, tumour mass is indicated by the brackets and the corresponding H&E stained sections are shown in (**g**,**h**).

Figure 4 shows the LA-ICP-MS data acquired from a section of FFPE human eye containing UM. Images showing the distribution of Cu, Zn and Fe are given along with intensity plots for line scans across the tumour.

## 3. Discussion

### 3.1. Lipid Metabolism in UM Patients

The finding that lipid species can still be observed in FFPE tissue in MALDI-MSI experiments is encouraging. For example, the LPC peaks (e.g., *m/z* 586.2, [M + K]^+^, LPC 20:2) are observable in these data sets and show some variation between patients. LPC is hydrolysed to produce lysophospatidic acid (LPA) by lysophospholipase D, otherwise known as autotaxin [10]. Previous studies of UM have shown that reduced expression of autotaxin (lysophospholipase D) correlates with a bad prognosis [11]. Thus, higher levels of LPC could correlate with lower levels of autotaxin expression and hence may be associated with a poor prognosis. It was also of interest that patients with genetic biomarkers indicative of a very poor outcome had less clustering of profile technical replicates, suggesting variation of profile readings (Figure 3). Increasing genetic instability is considered an enabling characteristic of cancers and is related to progression [12]. It is possible that those UM identified by genetic biomarkers as being more aggressive (Table 1), are more progressed, and have greater instability. As such, these UM could be more heterogeneous, and this may be reflected by the variation of repeat samples for these cases. Although this data is preliminary, coming from a small patient cohort, the observations made are scientifically consistent, and provide evidence to suggest that the species identified are of biological significance and can inform on the metabolic pathways active in individual UM.

### 3.2. Elemental Pathology of UM

Analysis of trace metals in other ocular diseases (such as age-related macular degeneration (AMD)) has included zinc, copper, cadmium, manganese, lead and mercury; each reported to have a potential role in disease pathogenesis [13,14,15,16,17]. However, there is a distinct lack of trace elemental research in the UM field. In addition to this, even diseases like AMD; studies have only observed trace metals within patient serum, plasma and the vitreous humour making tissue distribution analysis a very exciting prospect in UM [18]. As can be seen in Figure 4c,d, there appears to be a relative increase in ^63^Cu towards the periphery of the tumour in the poor prognosis patient. The link between copper and initiation of angiogenesis is well established through stimulation of proliferation and migration of endothelial cells, in addition, copper has been shown to impact factors involved in the initiation of angiogenesis such as VEGF and angiogenin [19,20,21]. Further to this, previous work has shown some evidence of the movement of copper to the extracellular space by endothelial cells and this may provide justification for the increase in peripheral copper as angiogenesis is being initiated, an important aspect for the growth and metastasis of tumours [22]. 

Zinc has been shown to be essential in tumour growth with cell proliferation being halted in the absence of this element [23,24]. Zinc, however, is required for the activity of hundreds of enzymes, and its increase in poor prognostic UM could signify high proliferation/metabolism and justify the apparent increase of intratumoural ^66^Zn (Figure 4e,f) [25]. Alternatively, both copper and zinc are important in the production of melanin, and as the poor prognostic UM was more pigmented than the good prognostic UM with lower levels of zinc, it is possible that these findings are related to the increased levels of melanin [26]. Given that copper is also important to melanogenesis, the findings of its restriction to the edge of the UM, and unrelated to pigmentation, may therefore be of more interest. Further investigation is necessary to make any solid link between trace element localisation and UM patient prognosis, and to ameliorate an atomic pathology pathway for trace elements and uveal melanoma.

### 3.3. Linking Tumour Metabolomic Heterogeneity to Clinical and Pathological Details

Solid tumour heterogeneity is well documented within the field of mass spectrometry imaging with advancements made to the visualisation, spectral segmentation and quantification of key molecular species [27,28]. Complex disease mechanisms continue to present a challenge when faced with continuous reprogramming of cancer cell metabolism, especially in rare yet aggressive malignancies like UM. Of interest in these preliminary imaging and PCA data are potential links between patient clinical and pathological details. With reference to Table 1 and Figure 3, the patients who were determined as having a very poor outcome by genetic biomarkers tended to have more variance between readings and less clustering of data when compared to the groupings and positioning of patients E and J with good prognosis alone found on the far-right half of the scores plot. 

## 4. Materials and Methods

### 4.1. Sample Preparation

A series of 11 enucleated eyes containing primary UM were investigated (Ethical approval STH ref: 15427, REC ref: 09/H1008/141). All patients had been treated over 7 years previously, and 6 UM were determined to have a poor prognosis by assessment of genetic biomarkers. Genetic biomarkers were determined using a combination of Fluorescence In Situ Hybridisation (FISH) and array comparative genomic hybridisation (aCGH) using the methods previously published [8,9]. Of the UM with poor prognosis all but 1 patient was confirmed as dead. The average survival of all patients was 51 months. 

Tissue fixation: The nucleations had been collected within a 6-year time period and were subject to the same fixation protocol, involving fixation in 10% buffered formalin for 24 h, dehydration in 70% EtOH and paraffin embedded. The 5 μm sections were cut using a microtome (Leica Microsystems, UK) and mounted onto a histological glass slide. FFPE tissue sections (*n* = 15) were stored at room temperature until further analysis.

Tissue preparation: Paraffin was removed from FFPE tissue sections by immersing the sample twice for 7 min in xylene substitute. The sections were gently hydrated for 4 min (×2) per solution in 100% EtOH, 95% EtOH and 70% EtOH, consecutively. Tissue was then submerged in 10 mM ammonium bicarbonate buffer for 5 min (×2). Heat induced antigen retrieval was then performed by immersing the tissue slide in a sealed tube containing 20 mM Tris-HCl buffer (pH = 9) and gradually brought to 97 °C for 45 min using a covered water bath. The section was cooled to room temperature, gently rinsed with tap water (1 min) to remove salt residues and then finally allowed to dry prior to matrix deposition. The antigen retrieval step, usually crucial for the successful detection of peptides was retained in the protocol to determine if small molecules still remained detectable by mass spectrometry within FFPE tissue samples that were prepared for immunohistochemistry using conventional methodologies.

### 4.2. MALDI Matrix Deposition

The matrix, CHCA in ethanol/water/TFA (3:1:0.1 by volume), was applied using a SunCollect pneumatic sprayer (SunChrom, Friedrichsdorf, Germany) (at 5 mg/mL) in a series of five layers. The first and second layers were sprayed at 3 μL min^−1^ to allow a matrix seeding process. Three subsequent layers were sprayed at 3.5 μL min^−1^ in a fine spray to ensure sample uniformity.

### 4.3. Instrumentation

Waters MALDI SYNAPT G2 HDMS–MALDI data were obtained using a Waters MALDI SYNAPT G2 HDMS mass spectrometer (Waters Corporation, Manchester, UK) to acquire mass spectra and images. Prior to MALDI-MSI analysis the samples were optically scanned using a flatbed scanner to produce a digital image for future reference, this image was then imported into the MALDI HDI software (Waters Corporation) to define the region to be imaged. The instrument was calibrated prior to analysis using phosphorus red. The instrument was operated in V-mode and positive ion mode, sensitivity mode (10,000 mass resolution), all data was acquired in the mass range *m/z* 100–1000 and the variable repetition rate Nd:YAG laser was set to 1 kHz. Image acquisitions were performed at 150 × 150 µm pixel size and 50 × 50 µm pixel size. The data was then converted and visualised using the High Definition Imaging (HDI) 1.1 software, (Waters Corporation, Manchester, UK); specificity type—IMS MS; number of most intense peaks—1000; resolution—10,000; low energy intensity threshold—50 (the low intensity threshold was to allow low abundant species to be included).

LA-ICP-MSI: Elemental analysis data were acquired using a Perkin Elmer NexION 350X inductively coupled plasma mass spectrometer (PerkinElmer Inc., Shelton, CT, USA) coupled to a New Wave UP-213 laser ablation chamber with Nd:YAG laser (New Wave Research, Inc., Fremont, CA, USA). Laser ablation parameters laser energy, spot size and scan speed were optimised and chosen to secure good spatial resolution without ablation of the glass slide underneath the section but maximising counts. For whole enucleated globe imaging, all samples were ablated using a line-by-line scan method, with laser lines drawn using the New Wave laser ablation kit software leaving 150 μm between each line. (This left space for another run for reuse of irreplaceable primary samples.) The quadrupole mass analyser was set to scan for *m/z* values 13, 56, 63 and 66 and had a scan time of 0.3 s. For tuning of the instrument, this was achieved daily using a predetermined tuning program provided by the PerkinElmer Syngistix software, using NIST 612 standard reference material of known trace elements in a glass disc and PerkinElmer’s own predetermined tuning programme. CPS data was recorded line by line each having its own comma separated. xl format. The IGOR PRO plugin was used in the iolite software to separate CPS recordings for each mass of interest and time to resolve them. A data reduction scheme in the software was used to produce the heat maps in a time and spatially resolved manner. All these images were normalised using C13, standard practice for bioimaging soft tissues due to homogenous spread in biological samples. Initial optimisation of the instrument settings was carried out using resected tumours and enucleated globe samples and plotting counts per second against time for individual line scans, at each setting. 

### 4.4. Haematoxylin and Eosin Staining

Slides from serial sections were immersed in haematoxylin for 1 min, rinsed in tap water until the water ran clear, immersed in 1% eosin for 30 s and rinsed in tap water until the water ran clear. They were then dehydrated as follows: 50% ethanol for 2 min, 70% ethanol for 2 min, 80% ethanol for 2 min, 95% ethanol for 2 min and 4 changes of xylene applied to each slide for 1 min at a time. Finally, they were mounted with DPX mountant (Sigma Aldrich, Dorset, UK) and left to dry in the fume hood overnight.

### 4.5. Statistical Analysis Using MALDI

Principle component analyses (PCA) were performed using MATLAB^®^ (Matrix Laboratory) (MathWorks, Inc., Natick, MA, USA) in conjunction with the Eigenvector PLS_Toolbox. The PCA statistics are representative of the UM data using six technical spectral repeats per biological sample (*n* = 11). The technical spectral replicates (six per biological sample) were acquired manually using MALDI-MS from the tumour regions of each eye section and MS results were then imported into MATLAB^®^ in.txt format after application of ‘automatic peak detection’ to achieve centroidal peak information using the instrument data processing software (WatersMassLynxTM Software). Normalisation (2-Norm) and mean centre were selected and ‘contiguous blocks’ were used for cross-validation.

## 5. Conclusions

In our study we were able to analyse simultaneously the different tissues of the eye and make some initial correlations with tumour behaviour and prognosis. Further analysis on a larger series of UM with more interrogation of the data is required to confirm the relevance of metabolite variation as determined by MALDI and trace metal analysis using LA-ICP-MSI. 

## Figures and Tables

**Table 1:** Clinical pathological details of the 11 patients.

Patient	Clinical and Pathological Details	Survival	Chromosomes 3 and 8 Status Determined by FISH	aCGH	Prognosis Determined by Genetic Biomarkers
A	Choroid. Diameter 13.8 mm	Alive at 110 months	M3		Poor
B	Ciliary body, spindle cell type, diameter 20 mm	Died metastatic disease 131 months	D3	Partial loss 3p and 3q, 6p+, −9	Good/intermediate
C	Choroid mixed cell type, diameter 16.4 mm	Alive at 52 months	D3		Good/intermediate
D	Choroid, spindle cell B, diameter 14.9 mm	Died unknown cause 47 months	M3		Poor
E	Choroid epithelioid cell type, diameter 11.1 mm	Alive 57 months	D3		Good
F	Choroid mixed cell type, diameter 12.5 mm	Died metastatic disease 38 months	M3 8q+		Very poor
G	Ciliary body spindle B, diameter 10.16 mm	Alive 120 months	D3	D3, partial 8q+, 1p-, partial 2p+ 6p+, partial 11p+	Good/intermediate
H	Ciliary body spindle, diameter 14.7 mm	Died metastatic disease 38 months	M3 8q+	M3, 8q+ and partial 1p-,−2,−13,	Very poor
I	Ciliary body/choroid, epithelioid cell type, diameter 6 mm	Died metastatic disease 32 months	M3 8q+	M3 8q+ and 1q+,4p+, 6q, −9 −10,−13,−15, 16q-	Very poor
J	Choroid, spindle B cell type, diameter 10.3 mm	Alive at 3 months	D3		Good
K	Choroid, spindle B cell type, diameter 13.6 mm	Died metastatic disease 39 months	M3, 8q+	M3, 8q+ and i(1q), partial 6q-	Very poor

## Data Availability

The data presented in this study will be made available at the Sheffield Hallam University Research Data Archive SHURDA and will be found at https://shurda.shu.ac.uk.
